# Late-Onset Nonhereditary Cherubism: First Reported Case in Ghana With Review of Diagnostic and Management Challenges

**DOI:** 10.1155/crra/5333205

**Published:** 2025-10-08

**Authors:** Seth Kwadjo Angmorterh, Mariella Mawunyo Amoussou-Gohoungo, Adam Inusah, Bridgette Palm, Kafui Kossi Kekessie, Riaan van de Venter, Sonia Aboagye, Cosmos Yarfi, John Nsor-Atindana, Portia Mamle Angmorterh, Klenam Dzefi-Tettey

**Affiliations:** ^1^Department of Medical Imaging, School of Allied Health Sciences, University of Health and Allied Sciences (UHAS), Ho, Ghana; ^2^Radiology Department, Greater-Accra Regional Hospital, Accra, Ghana; ^3^Department of Radiography, Faculty of Health Sciences, School of Clinical Care and Medicinal Sciences, Nelson Mandela University, Gqeberha, South Africa; ^4^Department of Speech, Language & Hearing Sciences, School of Allied Health Sciences, University of Health and Allied Sciences (UHAS), Ho, Ghana; ^5^Department of Physiotherapy and Rehabilitation Sciences, School of Allied Health Sciences, University of Health and Allied Sciences (UHAS), Ho, Ghana; ^6^Department of Nutrition and Dietetics, School of Allied Health Sciences, University of Health and Allied Sciences (UHAS), Ho, Ghana; ^7^Department of French Education, Faculty of Foreign Languages Education, University of Education, Winneba, Ghana; ^8^Department of Radiology, School of Medicine, University of Health and Allied Sciences (UHAS), Ho, Ghana

**Keywords:** cherubism, cherub-like face, craniofacial fibrous dysplasia, mandible swelling, monostotic disease, multilocular cystic disease, nonfamilial cherubism, nonhereditary cherubism

## Abstract

**Background:**

Cherubism is a rare genetic disorder characterised by multilocular cystic lesions in the mandible and/or maxilla, which result in the typical cherub-like face. Two forms of cherubism exist—hereditary (familial) and nonhereditary (nonfamilial)—and it usually occurs amongst children aged 2–7 years. The disorder is caused by a mutation in the SH3BP2 gene on Chromosome 4p16.3, essential for jaw development. The prognosis of cherubism shows that lesions increase in size and plateau at puberty, after which the lesions begin to regress and become undetectable. We present the first case of cherubism to be reported from Ghana.

**Case Presentation:**

Our case is a nonhereditary (nonfamilial) cherubism in a 21-year-old Ghanaian woman. The patient presented with bilateral asymmetrical facial swelling, jaw pain, trismus, toothache, tooth mobility and tooth loss. Also, the patient had facial disfigurement, weight loss, cough, headache, seizures and dizziness. Her family history was noncontributory. The results of a head computed tomography (CT) scan indicated chronic left sphenoid sinusitis and an enlargement of the entire mandible, with multilocular expansile lytic (soap bubble) appearance. The mandibles had ground glass matrix areas associated with subtle cortical destruction and dental deformities suggestive of Grade III cherubism.

**Discussion and Conclusion:**

Our patient had the onset of the disorder at age 12 but presented to the hospital at the age of 21. The reason why our patient did not seek medical help beforehand could be attributed to sociocultural beliefs, financial constraints and/or limited access to healthcare amenities. Treatment protocols for cherubism may include observation, surgery and medical therapy. Our patient resorted to the use of traditional medicine and spiritual/religious consultations for treatment. Our patient experienced discrimination due to the disease and had lost job, friendship and romantic relationship opportunities because of cultural beliefs, stereotypes and stigmatisation. Her facial disfigurement and the deformities are associated with bad omens and negative spirits in Ghana. When diagnosed, patients suffering from cherubism must be encouraged to seek information, education and appropriate individualised evidence-based management from hospitals. Treatment of the disease should be supported with psychological counselling and community sensitisation wherever possible.

## 1. Introduction

Cherubism is a rare genetic disorder characterised by multilocular cystic lesions in the mandible and/or maxilla that result in severe deformities [1–3]. Whilst cherubism shares some histological features with fibrous dysplasia (FD), it is a distinct genetic disorder caused by mutations in the SH3BP2 gene, primarily affecting the jawbones. Specifically, bilateral involvement of the posterior part of the mandible causes the development of the cherub-like facial appearance [4]. Two forms of cherubism exist—hereditary (familial) and nonhereditary (nonfamilial) [5]. According to Morice et al. [6], approximately 400 cases of cherubism have been documented globally. Currently, there are no documented statistics for cherubism in Africa. We present a case of late-onset, nonhereditary cherubism in a 21-year-old Ghanaian woman. This is the first reported case of cherubism in Ghana that we are aware of.

## 2. Case Presentation

### 2.1. Clinical History

**Figure 1:**
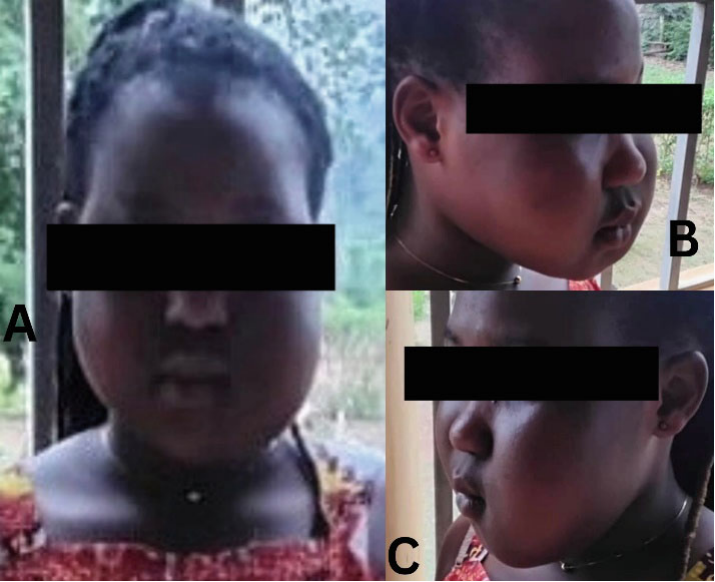
(A) Anterior view of the patient showing bilateral asymmetrical swelling of the mandibles. (B) Right and (C) left swelling of the patient's mandibles giving an appearance of cherubism.

A 21-year-old female patient was referred to the radiology department at the Ho Teaching Hospital, Ho, Ghana, with a history of bilateral facial swelling (Figure 1). The swelling began at age 12 in the left mandibular region and progressively increased in size. At age 21, she reported to the hospital after noticing a similar progressive swelling on the right side of her face. Her family history was noncontributory.

### 2.2. Dental History

Our patient's clinical history revealed that she experienced jaw pain, trismus, toothache, tooth mobility, tooth loss, facial disfigurement and headache.

### 2.3. Physical Examination Findings

Physical assessment revealed facial asymmetry and bilateral facial mass. The mass involved the left and right buccal, mandibular, submandibular and submental regions, with enlarged right anterosuperior cervical lymph nodes. There was no history of prior imaging. A head computed tomography (CT) scan was requested.

### 2.4. Imaging Findings

**Figure 2:**
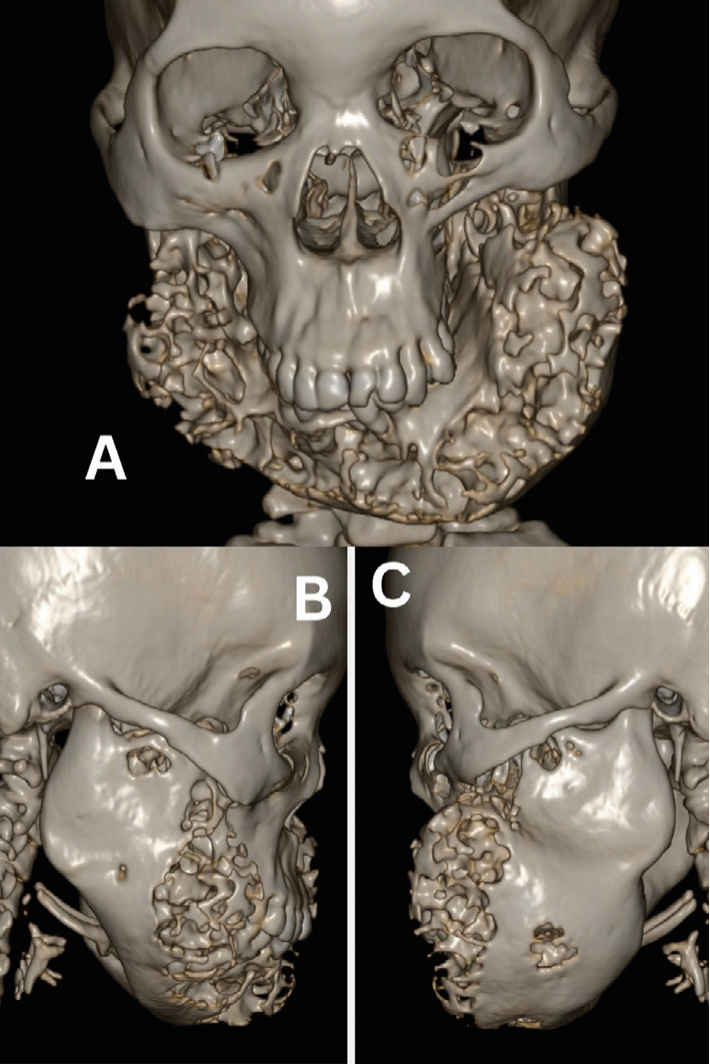
3D volume-rendered CT images of the patient's face. (A) Frontal view showing bilateral mandibular swelling. (B) Right mandibular swelling. (C) Left mandibular swelling. The findings are consistent with the characteristic appearance of cherubism.

**Figure 3:**
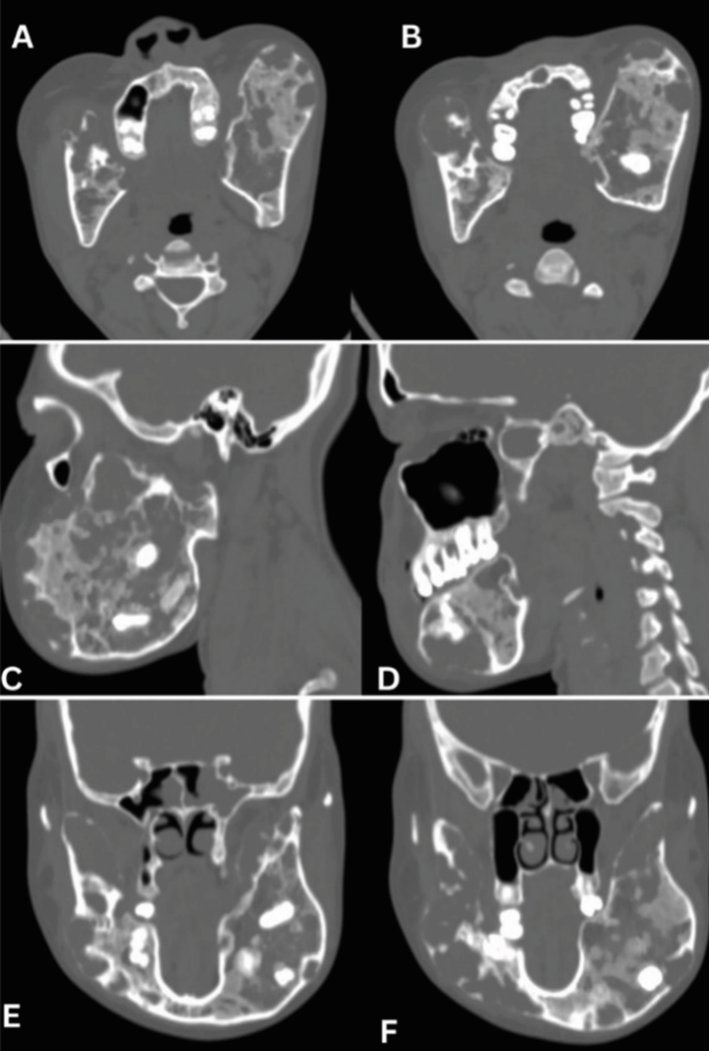
Multiplanar reformatted CT scan of the mandibles. Bone window showing multilocular expansile lytic (soap bubble appearance) and ground glass matrix areas associated with subtle cortical destruction suggestive of cherubism with (A, B) dental cavities, (C) atypical tooth orientation, and (D–F) tooth root resorption.

The multiplanar reformation (MPR) CT reconstructions of the head revealed normal CT features of the cerebral parenchyma. There was near-total opacification of the left sphenoid sinus with remodelling/thickening of the walls of the sinus indicative of chronic left sphenoid sinusitis. The pituitary fossa, orbits, mastoid air cells and remaining paranasal sinuses were normal. However, a mixed density lesion was observed, with severe expansile enlargement of the bilateral mandibles, exhibiting multilocular (soap bubble) and lytic cystic areas, as well as ground glass matrix changes with subtle cortical destruction, suggestive of cherubism (Figures 2 and 3). There was no associated soft tissue mass, fracture or periosteal reaction, and no maxillary involvement was noted. Although areas of cortical destruction raised concern for possible sarcomatous change, histopathological confirmation was not obtained.

### 2.5. Summary Diagnosis and Further Care

The summary diagnosis was Grade III cherubism. The patient did not proceed with further care in the hospital due to spiritual reasons.

## 3. Discussion

Cherubism affects the mandible and the maxillae and usually occurs amongst children aged 2–7 years [7, 8]. The age of onset of cherubism contradicts that of our case. Our patient had the onset of the disorder at age 12 but presented to the hospital at the age of 21. The reason why our patient did not seek medical treatment beforehand could be attributed to several factors including sociocultural beliefs, financial constraints and/or limited access to healthcare amenities. For example, in Ghana, health-seeking behaviours are often influenced by spiritual and cultural beliefs because some illnesses are considered by society and some patients to have spiritual/traditional connotations [9–11]. Therefore, to resolve these disorders and health conditions, traditional health practitioners are first consulted leading to delays in seeking hospital care. Additionally, the high cost of healthcare interventions compared to income levels in Ghana, especially those involving the use of advanced medical imaging like CT scans, which are not covered by the National Health Insurance Scheme (NHIS), can lead to patients delaying or avoiding hospital visitations altogether [12]. Finally, the inadequate distribution of hospitals and radiological equipment in Ghana, resulting in long patient waiting times, particularly in publicly owned hospitals, can also discourage patients from seeking timely care [13–15].

Across the two main types of cherubism—hereditary (familial) and nonhereditary (nonfamilial)—the familial type accounts for 80% of cases [16]. In both instances, the disorder is caused by a mutation in the SH3BP2 gene on Chromosome 4p16.3, essential for jaw development [3, 7, 16, 17]. Our patient had no remarkable family history of the disease, and therefore, her condition is a classic case of nonfamilial cherubism. It must be stated that no genetic test was performed on our patient due to the unavailability of the test within our hospital and the patient could not afford to have it performed in another hospital. In addition to genetic factors, mesenchymal alterations during jaw development, an odontogenic origin, hormonal and traumatic factors are possible causes of cherubism [18].

The progressive swelling of the face with a marked increase in fullness of cheeks and jaws is common to cherubism, and it is due to enlargement and expansion of the underlying bony structures, the skin and subcutaneous tissues. Cherubism has a predilection for males, with a ratio of 2:1, and frequently affects the mandible [3]. Although our case involved a female, the authors cannot conclude that cherubism has a predilection for females across the Ghanaian population because this is, to our knowledge, the first case of cherubism to be reported from Ghana. To determine the prevalence of the disorder in Ghana, further studies would be required. Literature on the prognosis of cherubism shows that lesions increase in size and number and plateau at puberty [19]. Afterwards, the lesions begin to regress after puberty, fill with bone and remodel until around 30 years of age when they often become undetectable [20–22]. According to Sonpal et al. [16], the remission of the jaw lesions could be attributed to the increased synthesis of sex steroids during puberty which may counteract the genetic anomaly, thereby mitigating localised osteoclast proliferation in cherubism. The prognosis of the disease in our patient is contrary to the evidence in literature. The disorder started in our patient at age 12 on the left half jaw, but instead of plateauing, it is progressing aggressively and has affected the right half jaw at age 21. Given the atypical course of the disease in our patient, other differential diagnoses such as FD variants (monostotic FD, polyostotic FD and McCune–Albright syndrome), giant cell lesions and sarcoma were considered.

As shown in Table 1, the grading system for cherubism encompasses the anatomical involvement, aggressiveness of the disease and mutation of SH3BP2 inheritance [23, 24]. The disorder presented in our patient reflects that of Grade III because, as shown in Figure 3d, the CT findings of our patient showed aggressive multilocular ill-defined expansile lytic and ground glass matrix lesions with tooth root resorption.

The diagnosis of cherubism depends on a combination of the clinical presentations, family history, radiographic findings, histological evidence and molecular genetic testing [7, 17, 25]. Radiographically, cherubism can be detected between the ages of 18 months and 2 years [17]. The radiographic features of cherubism change during the course of the disease. Medical imaging, in particular CT, magnetic resonance imaging (MRI) and orthopantomography (OPG) provide valuable information in the diagnosis of cherubism. For example, as seen in our case report (Figures 2 and 3), CT bone window enables the evaluation of strong densitometric alterations of the affected bone, and these images could range from sclerotic bone alterations to localised osteolytic processes, depending on the degree of mineralisation [26, 27]. Magnetic resonance imaging offers superior soft tissue characterisation and may better delineate marrow or cystic changes [7, 28]. On both CT and MRI images, cherubism may present as a multilocular, expansive, and/or nondestructive mass, with a well-circumscribed cortical and ground glass appearance [29–31]. Unfortunately, MRI services are very expensive and not easily accessible in Ghana [15], and therefore, an MRI scan was not performed on our patient.

Cherubism is usually associated with buccal cavity abnormalities such as toothache, tooth mobility, atypical dental arch and tooth eruption, progressive gingival enlargement, tooth resorption, teeth loss and malocclusion [3, 4, 17, 32, 33]. The disease is also associated with difficulty in chewing [34]. Our patient experienced trismus, toothache, tooth mobility, tooth loss and tooth root resorption. Similarly, Stoor et al. [7] indicated that abnormal dentition, premature loss of deciduous teeth and widely spaced, displaced and unerupted permanent teeth are key dental features of cherubism. As evidenced in our patient, cherubism may lead to conditions such as sinusitis which could cause upper airway obstruction, headaches and cervical lymphadenopathy [1, 4, 6, 16, 33–35]. The negative impact of cherubism goes beyond physical. According to Bhattacharya and Mishra [3], the disorder often causes severe psychological distress to patients due to the obvious facial deformities associated with the disorder. The psychological effects could be severe in African settings such as Ghana where facial disfigurement and deformities are associated with stigmatisation, bad omen and negative spiritualities [36]. For instance, our patient experienced social exclusion, low self-esteem, psychosocial distress and poor quality of life due to the stigma of the disease. Therefore, to ensure effective management of cherubism, psychological counselling and community sensitisation should accompany clinical care.

Treatment protocols for cherubism are personalised and not well established. However, the widely accepted treatment for cherubism is observation, as the disorder often resolves spontaneously [37]. Surgery may be considered if the deformity is associated with impaired breathing and swallowing, interference with tongue movement or compression of adjacent tissues with nerve constriction [1, 17]. However, surgical treatment could have two main disadvantages—risk of relapse (especially during the rapid growth phase of the disease) and pathological fracture [38–42]. In some patients, medical therapy in the form of calcitonin, bisphosphonates and calcineurin inhibitors can also be used in the management of cherubism [32, 43, 44]. Our patient resorted to the use of traditional medicine and spiritual/religious consultations for treatment. As noted earlier, this approach is common amongst Ghanaians; as many believe, some disorders have spiritual or traditional causes and consult traditional health practitioners, whose advice is often perceived as divinely inspired [9–11].

## 4. Conclusion

We report the first documented case of cherubism in Ghana. Cherubism is a rare benign bone disorder of the jaw that warrants the attention of health professionals in its diagnosis and management. Advanced radiology modalities such as CT can be useful in the precise diagnosis and the evaluation of the extent of the lesion. Treatment protocols for cherubism may include observation, surgery and medical therapy. Our patient resorted to the use of traditional medicine and spiritual/religious consultations for treatment. Our patient experienced discrimination due to the disease and lost job and relationship opportunities as a result because facial disfigurement and deformities are associated with bad omens and negative spirits in Ghana. When diagnosed, patients suffering from cherubism must be encouraged to seek for information and appropriate individualised evidence-based treatment and/or management from hospitals, supported by psychological counselling wherever possible.

### 4.1. Limitations

• Although sarcomatous change was suspected, biopsy was not done to exclude malignancy due to financial constraints.
• Genetic testing was not performed due to unavailability within our hospital, and the patient could not afford to have it performed in another hospital.

## Figures and Tables

**Table 1:** Grading system for cherubism [23, 24].

**Grade**	**Definition**
0	Existence of the mutation without expression of the disease
I	Lesion of the mandible without signs of root resorption
II	Lesions involving the mandible and maxilla without signs of root resorption
III	Aggressive lesion of the mandible with signs of root resorption
IV	Lesions involving the mandible and maxilla with signs of root resorption
V	The rare, massively growing, aggressive and extensively deforming juvenile lesions involving the maxilla and mandible
VI	The rare, massively growing, aggressive and extensively deforming juvenile lesions involving the maxilla, mandible and orbits

## Data Availability

The data used to support this case report, including radiological images and clinical details, are available within the article. Additional data can be made available by the corresponding author upon request.

## Nomenclature

CT: Computed Tomography
FD: Fibrous Dysplasia
MPR: Multiplanar Reformation or Reconstruction
MRI: Magnetic Resonance Imaging
NHIS: National Health Insurance Scheme
OPG: Orthopantomography
USA: United States of America
